# Multi-omics analyses were combined to construct ubiquitination-related features in colon adenocarcinoma and identify ASNS as a novel biomarker

**DOI:** 10.3389/fimmu.2024.1466286

**Published:** 2024-10-09

**Authors:** Zhaohui Wang, Wenbing Zhang, Xin Yin, Qinqing Wu, Yongwei Zhang, Yeben Qian, Qian Bao, Fubao Liu

**Affiliations:** ^1^ Department of General Surgery, The First Affiliated Hospital of Anhui Medical University, Hefei, China; ^2^ Department of General Surgery, Anqing First People’s Hospital of Anhui Medical University, Anqing, China; ^3^ Department of Preventive Medicine, Shantou University Medical College, Shantou, China; ^4^ Department of Pediatric Cardiac Surgery, Beijing Anzhen Hospital, Capital Medical University, Beijing, China

**Keywords:** colon adenocarcinoma, prognostic signature, single-cell transcriptome sequencing, ASNS, immunotherapy

## Abstract

**Background:**

As one of the malignant tumors with the highest incidence and fatality in the world, colon adenocarcinoma (COAD) has a very complex pathogenic mechanism, which has not yet been fully elucidated. Ubiquitin can regulate cell proliferation, cell cycle, apoptosis, DNA damage repair, and other processes by changing the activity of substrate proteins or causing ubiquitin-proteasome degradation. These are the key links in the pathogenesis of COAD, and ubiquitin plays an important role in the occurrence and development of COAD.

**Methods:**

We integrated transcriptomics, single-cell and clinical omics, and TCGA and GEO databases of COAD patient data. Cox and Lasso regression was employed to assess ubiquitination genes in COAD for generating ubiquitination-related features. The aim was to evaluate the prognostic value of these features for tumors and their impact on the immune microenvironment. At the same time, the expression level of model genes was further analyzed using single-cell data. Finally, the expression and function of ASNS, a key gene for this trait, were detected *in vitro*.

**Results:**

In our study, based on identifiable changes in the expression of marker genes, this feature can be used to classify patients with COAD. Kaplan-Meier survival analysis indicated that those with elevated risk scores in each cohort experienced inferior outcomes. There is good validation in both the training queue and the validation queue. The results of the immune infiltration analysis showed that the immune infiltration rate was significantly increased in the high-risk group. After the knockdown of ASNS, an important gene in the signature, the activity and migration capacity of SW620 and RKO cell lines and colony formation capacity were dramatically reduced in cell tests.

**Conclusion:**

We screened ubiquitination-related genes and constructed ubiquitination-related features, which can be used as reliable prognostic indicators of COAD. ASNS was identified as a possible biomarker for COAD.

## 1 Introduction

As per the GLOBOCAN 2022 report from the International Agency for Research on Cancer (IARC), colon adenocarcinoma holds the third position in global cancer incidence, following lung cancer and female breast cancer. In terms of mortality, it ranks second globally, trailing only lung cancer in the spectrum of cancer-related deaths (1, 2). Surgical excision, radiotherapy, chemotherapy, targeted therapy, and immunotherapy can significantly improve the treatment outcome of COAD patients, but the prognosis of patients with advanced COAD remains poor (3). Therefore, it is critical to explore the tumor microenvironment of colon adenocarcinoma and develop new biomarkers to aid in the prognostic assessment and treatment of COAD.

In eukaryotic systems, post-translational modifications like ubiquitination, phosphorylation, acetylation, and glycosylation play crucial roles in upholding the biological functions of proteins (4, 5). Ubiquitination, centrally involved in numerous cellular processes, influences functions such as cell proliferation, apoptosis, differentiation, and DNA replication repair (6). Cells regulate protein degradation through protein quality control (PQC) signaling pathways that recognize substrates and direct their refolding or removal, thereby avoiding the accumulation of abnormal proteins in the cell (7, 8). Dominating the degradation of misfolded proteins, the ubiquitin-proteasome system (UPS) stands as the primary pathway for protein breakdown, participating in over 80% of intracellular protein degradation. Wang et al. found that UBE2J1 inhibits colorectal cancer progression by promoting ubiquitination and degradation of RPS3 (9). Wang et al. found that immune-associated NRC-SOX9-4 promotes colorectal cancer progression by inhibiting YBX1 polyubiquitination and degradation (10). Consequently, it is prudent to investigate the role of ubiquitination in COAD (11, 12).

Within tumor cells, the processes of ubiquitination and deubiquitination play pivotal roles in orchestrating the metabolic reprogramming observed in cancer cells (13). The metabolic adaptation of cancer cells has been associated with the ubiquitination of various molecules, namely mTOR, AKT, AMPK, c-Myc, p53, NRF2, KRAS, and HIF (14–16). In addition, ubiquitination in cancer cells is also associated with autophagy (17). For example, polyubiquitination modification of the K63 junction of ULK1 complex and type III PI3K complex can promote the stability of the complex and thus promote the activation of autophagy. Of course, there are many other functions associated with ubiquitination/deubiquitination (18).

Single-cell transcriptomics and bioinformatics analysis play a key role in cancer research (19, 20). They combine high-throughput techniques to deeply explore the transcriptome characteristics of individual tumor cells and reveal the distribution of intra-tumor heterogeneity and cell subsets, contributing to the understanding of the mechanisms of cancer development, invasion, and metastasis (21). Bioinformatics analysis can process and interpret these massive data, helping us to deeply understand the biological characteristics of tumors at the global and cellular level, providing an important basis for precision medicine (22).

In this study, we combined bioinformatics analysis of COAD data from TCGA and GEO data to investigate the involvement of ubiquitination-related genes in COAD. Ubiquitination-related prognostic features were developed to classify COAD patients into high-low risk groups. Moreover, within the context of COAD, ubiquitin signatures offer a means to detect alterations in immune infiltration as well as immune checkpoint activity (23). Our investigation aims to enrich prognostic evaluations and facilitate the advancement of treatments for COAD.

## 2 Materials and methods

### 2.1 Data obtainability

In this research, scRNA-seq data for 23 COAD tumor samples and 8 normal samples were sourced from the GSE132465 database available on the GEO website. The training and validation cohort included RNA expression data and corresponding clinical details for COAD from the TCGA database and GEO datasets GSE39582 (https://portal.gdc.cancer.gov/). Furthermore, a scrutiny encompassed 2634 ubiquitination-associated genes (URGs) sourced from the GeneCards database, each demonstrating a correlation score greater than 3.

### 2.2 Data processing

The analysis commenced with a differential assessment to discern the variances in gene expression between tumor and normal samples in TCGA, subsequently culminating in the generation of a heatmap and a volcano plot. Subsequently, we combined the COAD samples from TCGA and GSE39582 into a merged cohort. The function normalize between arrays in R was employed to remove batch effects, and data were converted to a log2 scale before the analysis. The overall survival (OS) of the merged cohort was analyzed using a univariate Cox regression, identifying prognostically significant URGs at a p-value of less than 0.05.

### 2.3 Identification of ubiquitination-related molecular subtypes

Utilizing gene expression profiles, 1017 COAD samples were stratified into distinct molecular subtypes. This classification was conducted using the ConsensusClusterPlus package, which incorporates a K-means clustering algorithm to organize the samples into robust clusters, with the maximum number of clusters set at nine (maxK = 9). Employing the cumulative distribution function (CDF) curve analysis and the CDF delta area curve, the identification of the optimal number of ubiquitination-associated subtypes was undertaken, in addition to the generation of a consensus matrix heatmap to visually depict cluster affiliations. Further analysis was undertaken to explore the spatial distribution and relationship among the identified subtypes. Incorporated within this analysis were PCA and UMAP, furnishing a graphical portrayal of subtype dispersion across the samples. Additionally, differential expression of URGs and survival disparities among the subtypes were investigated using a heatmap for gene expression visualization and the ‘survival’ package for survival analysis, respectively.

### 2.4 Enrichment analysis

Utilizing R packages “clusterProfiler” and “org.Hs.eg.db,” the analysis of differentially expressed genes across various subtypes involved leveraging Kyoto Encyclopedia of KEGG, GO enrichment, and GSEA (24). Furthermore, ssGSEA was utilized to quantify enrichment scores related to immune cell infiltration and immune functions.

### 2.5 The establishment and validation of a prognostic risk signature were undertaken

Subjects with comprehensive clinical data were randomly divided into training and testing cohorts at a 1:1 ratio. Utilizing the R package “survival,” a univariate Cox regression analysis was conducted to pinpoint genes associated with prognosis (25). These genes formed the foundation for constructing a prognostic model linked to ubiquitination, employing Lasso and multivariate Cox regression methodologies (26). Post-construction, individuals were categorized into high- and low-risk groups depending on their median risk scores. Each individual’s risk score was calculated using a specific formula: $Risk\;score=\sum_{i=1}^{n}\beta i*expi$ , where expi represents the expression level of each URG, and βi denotes the respective gene coefficient within the signature. The Kaplan-Meier method was then used to examine overall survival (OS) differences between the two risk groups. Furthermore, the signature’s predictive accuracy was appraised with the ROC curve. Additionally, multivariate Cox regression analyses were performed in both cohorts to verify the risk score’s role as an independent prognostic marker (27).

### 2.6 Nomogram formulation

The creation of a nomogram involved integrating the risk score, age, and pathological stage as independent prognostic variables for evaluating the likelihood of overall survival (OS) at 1, 3, and 5 years (28). To appraise the nomogram’s predictive precision, we employed the ROC, calibration, and cumulative hazard curves.

### 2.7 Tumor immune characteristics

Utilizing the CIBERSORT algorithm, we computed the proportions of immune infiltrating cells in each COAD sample. Based on this data, we assessed the differences in immune cell expression across various risk groups, analyzed the correlations among immune cells, and examined their relationships with risk scores. Additionally, comparisons of tumor microenvironment (TME) scores were conducted using the R package “estimate” (29). The activation of immune checkpoints between the two groups was visualized using a barplot.

### 2.8 Therapies and drugs

We utilized the ‘oncoPredict’ package to compute the IC50 values for various chemotherapy drugs across the two patient risk groups, aiming to gauge their sensitivity to chemotherapy. Variations between subtypes were analyzed using the Wilcoxon test.

### 2.9 Processing scRNA-seq data and annotating cells

The cell types annotated within the GSE132465 dataset were derived from prior studies. We conducted quality control on the scRNA-seq data using the “Seurat” and “SingleR” R packages. We included cells that had less than 10% of mitochondrial gene expression, more than 200 overall genes, and genes that were expressed in at least three cells, with expression levels ranging from 200 to 7000, to ensure the data's quality remained high. Subsequent analyses were conducted using the “Seurat” R package. We identified the top 2000 highly variable genes (HVGs) and employed the top 15 principal components in conjunction with these HVGs (30). Dimensionality reduction and visualization were achieved through UMAP to classify each cell type. Distinct cell types, such as T cells, B cells, and epithelial cells, were identified based on marker genes (31, 32).

### 2.10 CellChat analysis

We employed CellChat to assess the primary signaling inputs and outputs across all cell clusters, utilizing CellChatDB.human as the reference database. Subsequently, we applied the netVisual_circle function to illustrate the relative strength of cell-cell communication networks among the different cell clusters.

### 2.11 qRT-PCR

The tissue specimens were provided by Anqing First People’s Hospital affiliated with Anhui Medical University and preserved at -80°C. Ten tissue pairs, including tumor tissue (T) and precancerous tissue (N), were collected from CORD patients who underwent colon tumor resection between May 2023 and May 2024. This experiment was performed according to the previous research. All primers, along with their precise sequences, were provided by Tsingke Biotech (Beijing, China) and are detailed in **Supplementary Table 1**.

### 2.12 Cell proliferation assay

For the assessment of cell proliferation, we employed the CCK-8; Vazyme, Nanjing, China). Cells were seeded at a density of 5×10^3^ cells per well in 96-well plates. Subsequently, the plate underwent a two-hour incubation in the absence of light at 37°C with 10 μl of CCK-8 labeling reagent per well. Cell viability was evaluated by measuring the absorbance at 450 nm using an enzymatic label reader (A33978, Thermo, USA) at intervals of 0, 24, 48, 72, 96, and 120 hours (33).

### 2.13 Wound healing

Upon reaching 95% confluency, the transfected cells were transferred to 6-well plates. A sterile pipette tip (200 μL) was used to create a straight line, followed by gentle rinsing with PBS to eliminate unattached cells and debris. The serum-free medium was then substituted to sustain cell culture. Images were taken at 0 and 48 hours in the identical position (34).

### 2.14 Colony formation

After transfecting 1000 cells, we incubated them in 6-well plates for about 14 days. At the end of this period, the cell clones became visible to the naked eye. Subsequently, the cells underwent a 15-minute fixation in 4% paraformaldehyde (PFA). Following this, staining with Crystal Violet (Solarbio, China) was conducted for 20 minutes, and the cells were air-dried at room temperature before being counted per well.

## 3 Results

### 3.1 Identification of subtypes of COAD based on ubiquitination-related genes.

**Figure 1:**
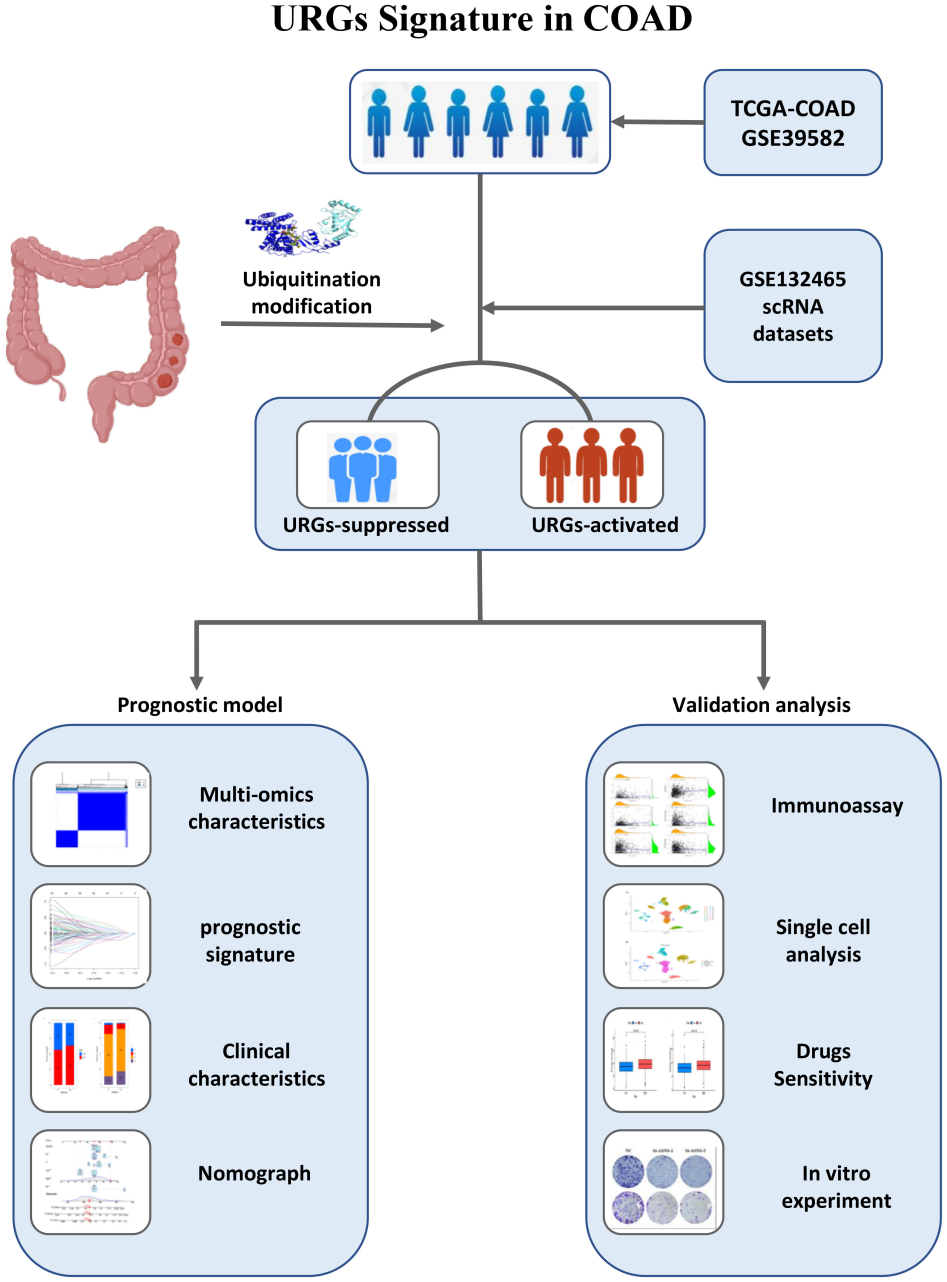
The flowchart of our study.

**Figure 2:**
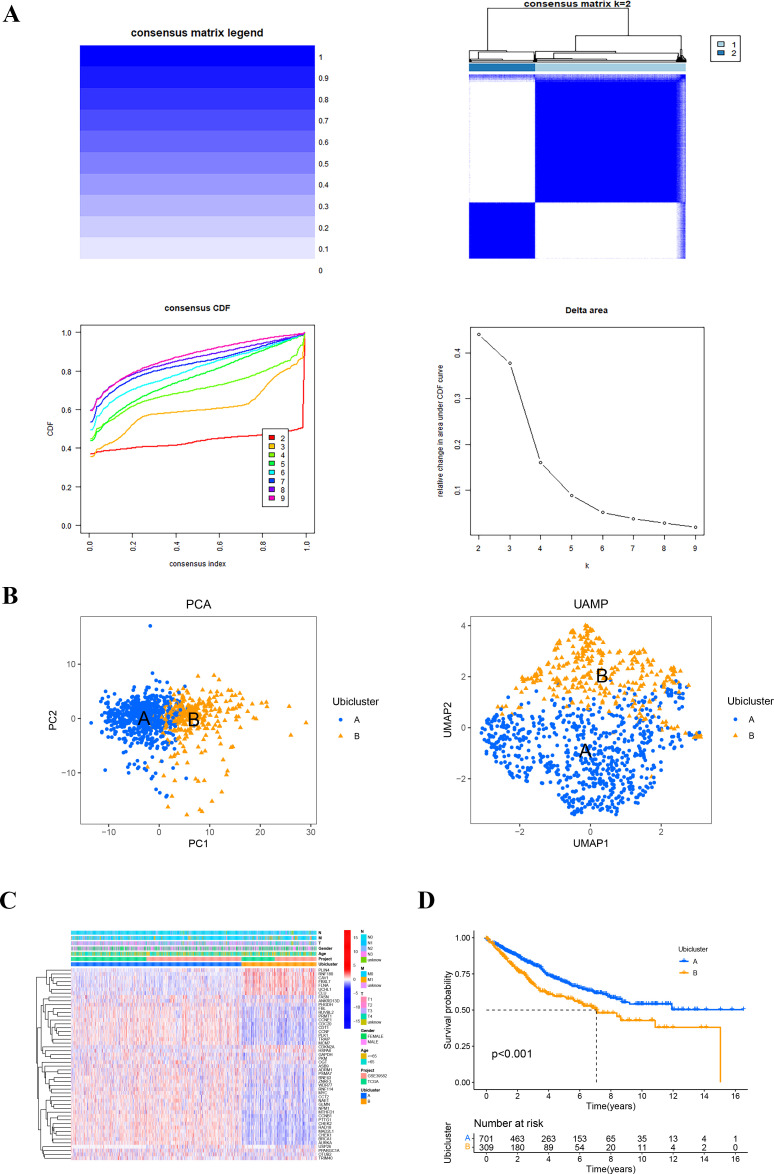
The ubiquitination-related molecular subtypes of COAD. **(A)** Consensus clustering was performed on 1017 samples from the TCGA and GEO databases based on genes related to ubiquitination. The cumulative distribution function (CDF) plot indicated that the curve remained relatively flat at K = 2. The relative change in the area under the CDF curve between K and K-1 showed a more pronounced slope change after K values of 2 and 3. Consequently, K = 2 was selected as the optimal number of clusters, and the consistency matrix was presented. **(B)** Principal component analysis (PCA) and uniform manifold approximation and projection (UMAP) diagrams illustrated the two clusters. **(C)** The expression heatmap of ubiquitination-related genes was shown for the two clusters, along with clinical features. **(D)** Survival curves for the two clusters were depicted.

The flowchart of our study is shown in **Figure 1**. We first screened 581 differential expressed genes (DEGs) from a set of ubiquitination-related genes **Supplementary Figures 1**, **2**. Using 137 ubiquitination-related prognostic genes derived from univariate Cox regression analysis, consensus clustering was employed to identify the optimal number of ubiquitination-related subtypes **Supplementary Figure 3**. The cumulative distribution function (CDF) diagram indicated that the CDF curve stabilized at K = 2, reflecting a robust clustering consistency. Additionally, the area under the CDF curve demonstrated notable slope alterations beyond the K values of 2 and 3. Consequently, K = 2 was selected as the most appropriate number of clusters (**Figure 2A**). Subsequently, 1017 colon cancer samples were segregated into two distinct clusters. Cluster A comprised 706 samples, while Cluster B included 311 samples. Dimensionality reduction techniques such as PCA and UAMP confirmed that the clusters occupied divergent positions and were distinctly separable, affirming the reliability of the clustering results (**Figure 2B**). Examination of gene expression and survival disparities between the clusters showed that ubiquitination-related genes were markedly upregulated in Cluster A (**Figure 2C**). Survival analysis further highlighted a significant difference in survival outcomes between the two subtypes (P < 0.001), with Cluster B exhibiting poorer survival (**Figure 2D**).

### 3.2 Enrichment analysis

**Figure 3:**
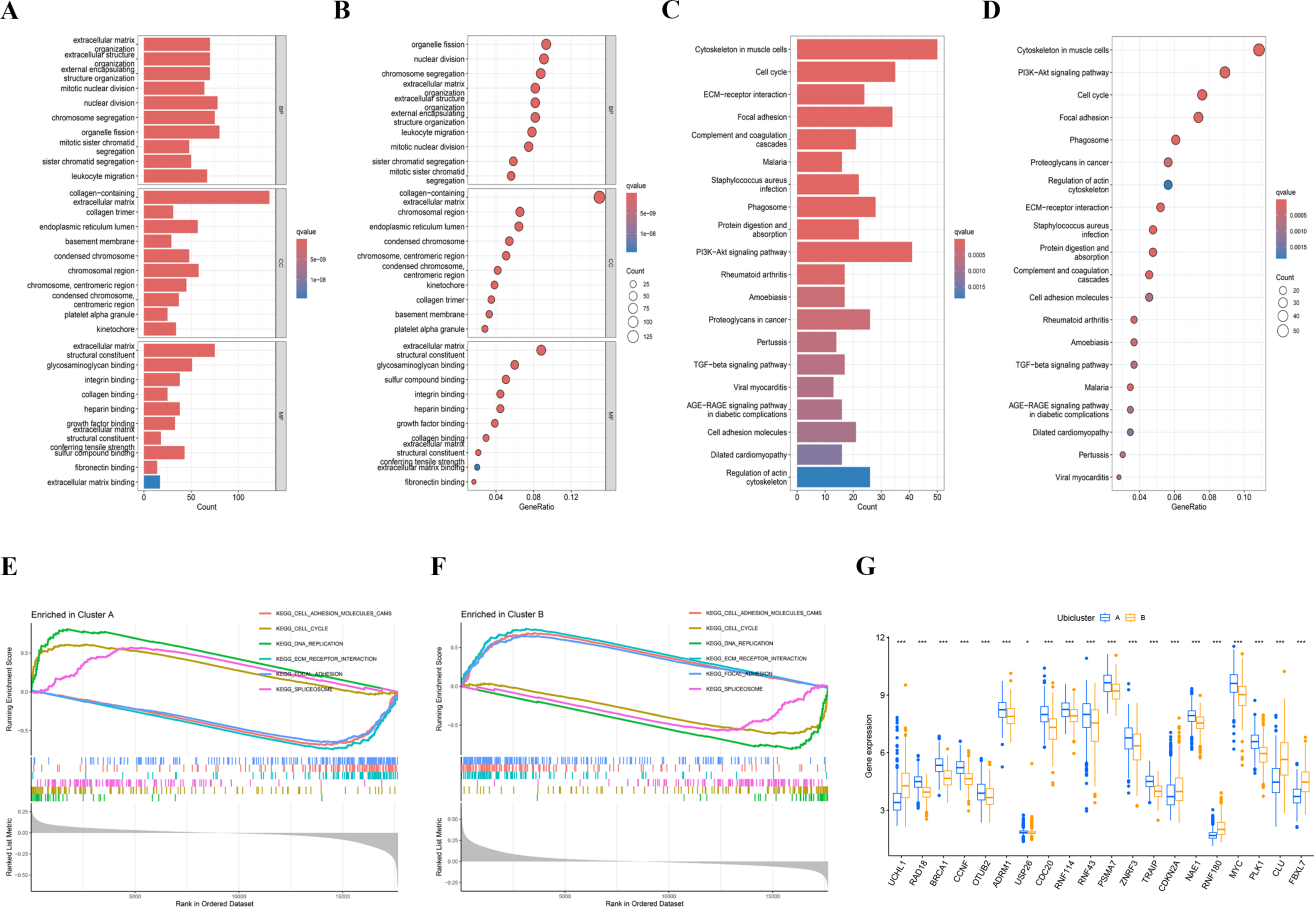
Enrichment analysis of different subtypes. **(A, B)** The Gene Ontology (GO) enrichment analysis results are presented in bar plots and bubble plots, categorized into biological process (BP), cellular component (CC), and molecular function (MF). The top five significant GO enrichment results are displayed. **(C, D)** The Kyoto Encyclopedia of Genes and Genomes (KEGG) enrichment results are shown using bar plots and bubble plots. **(E, F)** Gene Set Enrichment Analysis (GSEA) indicated that the cell cycle was particularly active in cluster **(A, G)** Single-sample Gene Set Enrichment Analysis (ssGSEA) scores for immune cells were compared between cluster A and cluster B groups, with higher ssGSEA scores observed in the cluster B.

Gene Ontology (GO) enrichment analysis was conducted on the DEGs, revealing their involvement in several biological processes (BP) such as nuclear division and organelle fission. Regarding cellular components (CC), they were primarily associated with the collagen-containing extracellular matrix and chromosomal region. Concerning molecular function (MF), the differentially expressed genes (DEGs) were predominantly linked to extracellular matrix structural constituent and glycosaminoglycan binding (**Figures 3A, B**). Importantly, the activity of glycosaminoglycan binding has been reported to be associated with the prognosis of colon adenocarcinoma. Concerning KEGG analysis, we found that differential genes are mainly enriched in cell cycle and focal adhesion pathways (**Figures 3C, D**). Additionally, Gene Set Enrichment Analysis (GSEA) was conducted on both clusters, leading to the identification of enriched pathways in cluster A, such as cell cycle, DNA replication, and spliceosome (**Figure 3E**). A comparison of hallmark pathway gene signatures between the two clusters revealed distinct patterns. In contrast, cluster B exhibited enrichment in ECM-receptor interaction, focal adhesion, and cell adhesion molecule pathways as the top three signatures (**Figure 3F**). The single sample gene set enrichment analysis (ssGSEA) scores of immune cells in group A and group B were compared. The ssGSEA scores in group B were higher, and the immune infiltration and risk scores were consistent (**Figure 3G**).

### 3.3 Establishment of a prognostic-related signature

**Figure 4:**
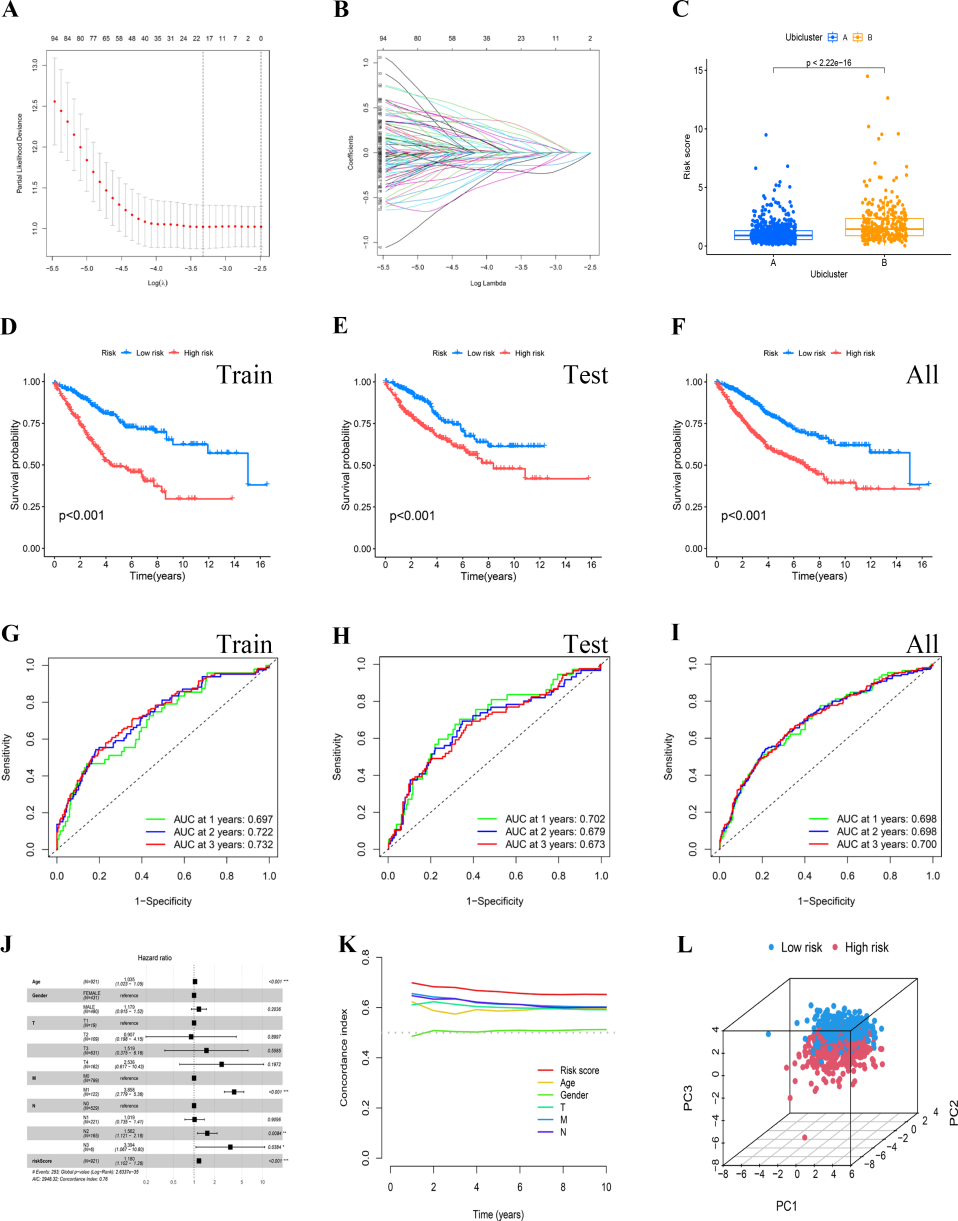
Establishment and identification of the risk signature. **(A, B)** The potential prognostic genes underwent LASSO-Cox regression analysis in the training cohort to develop a prognostic risk signature. **(C)** The risk scores for the two clusters were computed using the derived scoring formula. **(D–F)** To validate the reliability of the risk model, survival analysis was conducted between the high-risk and low-risk groups in the training cohort, the testing cohort, and the all cohort. **(H, I)** The ROC curves for patient survival over different years were plotted for the training, testing, and all cohorts. **(J)** Multivariate Cox regression analysis demonstrated that the risk score was an independent prognostic factor in BRCA patients. **(K)** The concordance index curve indicated that the risk signature offered strong predictive accuracy. **(L)** PCA analysis showcased the proficiency of the PRS in distinguishing COAD samples.

Initially, a univariate Cox proportional hazards regression analysis was conducted on a merged dataset from TCGA and GEO, identifying 137 ubiquitination marker genes significantly associated with overall survival (OS, P<0.05). These genes formed the basis of a prognostic signature. The most beneficial prognostic genes were selected using a LASSO Cox regression model, and a final set of 12 genes was established through multivariate Cox regression analysis (**Figures 4A, B**). A heatmap of model gene expression is shown in **Supplementary Figure 4**. The risk score was then calculated using the formula: “ (-0.815)*USP26 + (-0.193)*MYC + 0.345*OGT + (-0.408)*PRMT1 + 0.546*SNAI1 + 0.566*RPS17 + (-0.45)*RPN2 + (-0.394)*ACACA + 0.419*RNF112 + 0.503*ASNS + 0.333*MC1R + 0.46*FBXO39”. This scoring algorithm was also applied to the combined validation and overall datasets. Patients were categorized into high- and low-risk groups based on the median risk score. Notably, cluster B, as previously defined, also exhibits a higher risk score in this model (**Figure 4C**). Kaplan-Meier survival curves illustrated that individuals in the high-risk category experienced significantly worse overall survival (OS) compared to those in the low-risk group (P<0.05) (**Figures 4D–F**). The areas under the ROC curves for 1, 2, and 3 years were 0.697, 0.722, and 0.732 for the training cohort; 0.702, 0.679, and 0.673 for the testing cohort; and 0.698, 0.698, and 0.700 across all cohorts, respectively (**Figures 4G–I**). The independence of the risk score from clinical characteristics was confirmed through multivariate Cox regression analyses, as depicted in **Figure 4J**. The Multi-Cox regression analysis unveiled that solely age and the risk score stood as autonomous variables, wherein the hazard ratio for the risk score was determined to be 1.180 (P<0.05). The concordance index curve also underscored that the risk signature provided better predictive accuracy than other clinical features (**Figure 4K**). Furthermore, a three-dimensional scatter plot of PCA analysis illustrated the ability of the 12-gene Prognostic Risk Score (PRS) to discriminate COAD samples effectively, indicating its superior discriminatory power (**Figure 4L**).

### 3.4 Clinical phenotypes and Nomogram formulation

**Figure 5:**
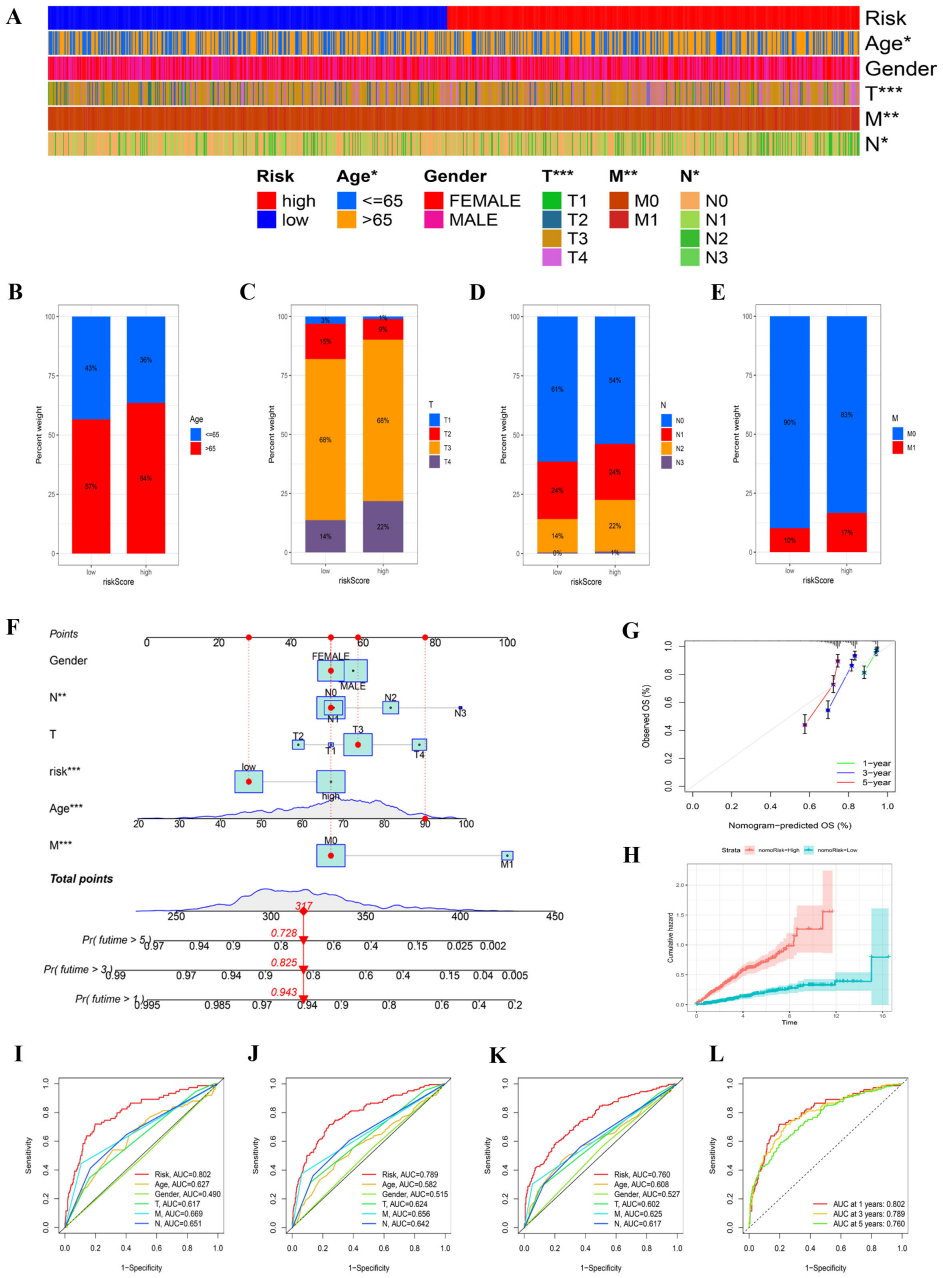
The clinical characteristics of two risk groups and the formulation of a nomogram. **(A)** A heatmap displayed the correlation between clinical factors and the risk groups. **(B–E)** The proportions of samples in the two risk groups were compared across various clinical data. **(F)** A nomogram was constructed by integrating clinical features with the risk score. **(G)** Calibration plots assessed the consistency between actual overall survival (OS) rates and predicted survival rates, with the 45° line indicating perfect prediction. **(H)** The cumulative hazard curve was used to evaluate the nomogram’s predictive performance. **(I–L)** ROC curves for 1, 3, and 5 years illustrated the AUC values for various clinical factors and nomogram scores.

In our effort to elucidate distinctions between risk groups, we analyzed clinical data from our merged cohort. A chi-squared test revealed significant differences in the distribution of COAD cohorts across age groups (P < 0.05) between varying risk groups, alongside notable variations in TNM stages (**Figure 5A**). Older age and more advanced TNM stages typified the high-risk cohort. To illustrate, 17% of individuals within the high-risk category were designated as stage M1, in contrast to only 10% in the low-risk subset (**Figures 5B–E**).

The construction of a nomogram aimed to assess patient risk by amalgamating clinical particulars with risk classification. **Figure 5F** provides a comprehensive overview of patient characteristics, including gender, age, T, N, and M stages, and risk classification. This predictive tool enables a more accurate estimation of patient risk and aids in formulating tailored therapeutic strategies (**Figure 5G**). The cumulative hazard curve indicates that patients with elevated nomogram risk exhibit a greater hazard (**Figure 5H**). To rigorously assess the predictive accuracy of the nomogram, a prognostic ROC analysis was performed, demonstrating superior performance compared to other clinical models and risk scores. The AUC values were 0.802, 0.789, and 0.760 at 1, 3, and 5 years, respectively (**Figures 5I–L**).

### 3.5 Immune analysis and therapy analysis

**Figure 6:**
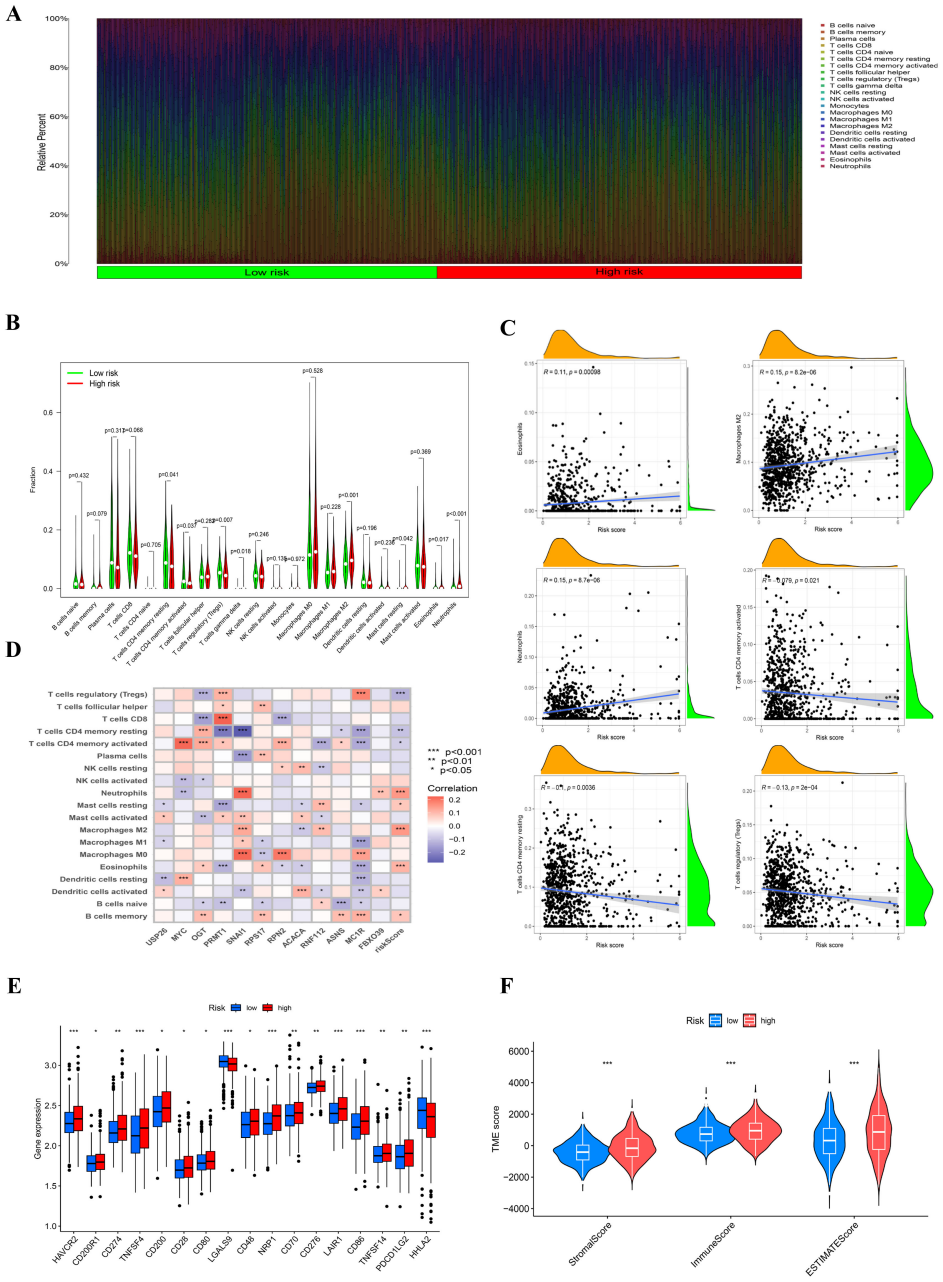
Analysis related to immune between two groups. **(A)** The proportions of immune cells in each sample were analyzed using CIBERSORT. **(B)** A violin plot compared the fractions of immune cells between the two subtypes, with statistical differences tested using the Wilcoxon test (P < 0.05). **(C)** The correlations between the risk score and various immune cell types were evaluated. **(D)** Correlations between immune cells/functions and risk signature genes were examined. **(E)** The expression levels of immune checkpoint genes differed between the two groups, with higher expression observed in the high-risk group (***P<0.001). **(F)** Immune-related scores, including stromal score, immune score, and ESTIMATE score, were compared between the two risk groups.

The figure illustrates that we used the CIBERSORT method to quantify and compare immune cell infiltration levels between the two risk groups employing the Wilcoxon test (**Figure 6A**). The violin plot presented in the study clearly demonstrates that there is a significant increase in the proportion of resting CD4 memory T cells within the low-risk group when compared to their counterparts in the high-risk group. This finding suggests a potential immune profile that is more favorable in the low-risk cohort, highlighting the importance of resting CD4 memory T cells in this context. Conversely, the high-risk group exhibited markedly higher levels of both M2 macrophages and activated mast cells, with statistical significance indicated by a p-value of less than 0.05 (**Figure 6B**). These observations can provide valuable insights into the differing immune landscapes present in varying risk categories. Furthermore, **Figure 6C** elaborates on the relationship between the risk score and the relative abundance of several immune cell types. It was observed that the risk score exhibited an inverse correlation with the presence of activated CD4 memory T cells, resting CD4 memory T cells, and regulatory T cells, indicating that as the risk score increases, the abundance of these beneficial T cell populations tends to decrease. In contrast, the risk score showed a positive correlation with the levels of eosinophils, neutrophils, and M2 macrophages, suggesting that higher risk scores are associated with an increase in these cell types. This differential distribution of immune cells based on risk scores points toward underlying mechanisms that may influence disease progression and patient outcomes.. In the model, the expression of the ASNS gene is inversely correlated with naive B cells, potentially indicating that the ASNS gene modulates changes in TME by regulating B cell proliferation (**Figure 6D**). We explored the relationship between risk scores and frequently identified immunotherapy biomarkers in the combined cohort. The analysis revealed that nearly all immune checkpoint genes (ICGs), including CD28 and CD70, were significantly overexpressed in the high-risk group (**Figure 6E**). The ESTIMATE methodology was employed to evaluate immune infiltration among various risk cohorts. The corresponding **Figure 6F** substantiated the preceding study, demonstrating that the high-risk cohort exhibited elevated estimate, stromal, and immune scores in comparison to another group.

### 3.6 Single-cell

**Figure 7:**
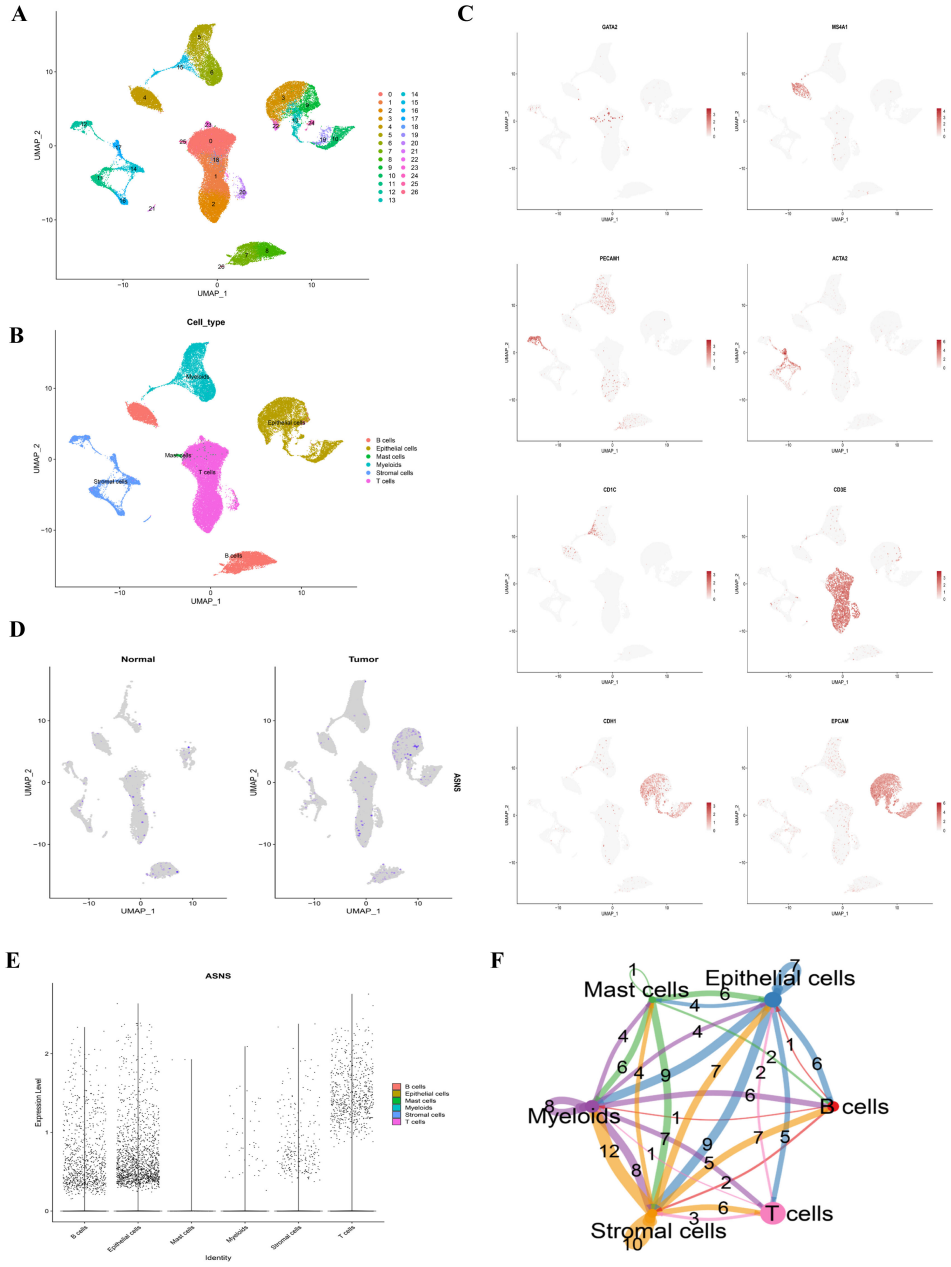
Single-cell classification and cell chat. **(A)** The UMAP plot displayed all the cells divided into 26 clusters. **(B)** Another UMAP plot indicated that COAD samples can be classified into 6 cell types. **(C)** Marker genes were identified for each cluster. **(D)** A feature plot illustrated the distribution of the gene ASNS across each cluster. **(E)** A violin plot depicted the expression levels of ASNS in each cluster. **(F)** Cell-cell communication between the six main cell types was analyzed using CellChat.

After processing and refining the data, gene expression profiles from 53844 cells of 12 COAD samples were gathered for further analysis. Employing dimensionality reduction and log-normalization, 26 distinct cell clusters were identified (**Figure 7A**). The characterization of cell types within each cluster was achieved by comparing differentially expressed genes with canonical markers (**Figure 7B**). Differential expression of marker genes was employed to differentiate between various cellular groupings, as illustrated in **Figure 7C**. We then studied the ASNS gene in the model in detail. In **Figures 7D, E**, it is evident that the ASNS gene is highly expressed in the epithelial cells of the tumor sample. In **Figure 7F**, epithelial cells and stromal cells have strong cellular communication, suggesting that ASNS genes may be involved in the interaction between epithelial and stromal cells to regulate the development of colon adenocarcinoma.

### 3.7 Drugs sensitivity

**Figure 8:**
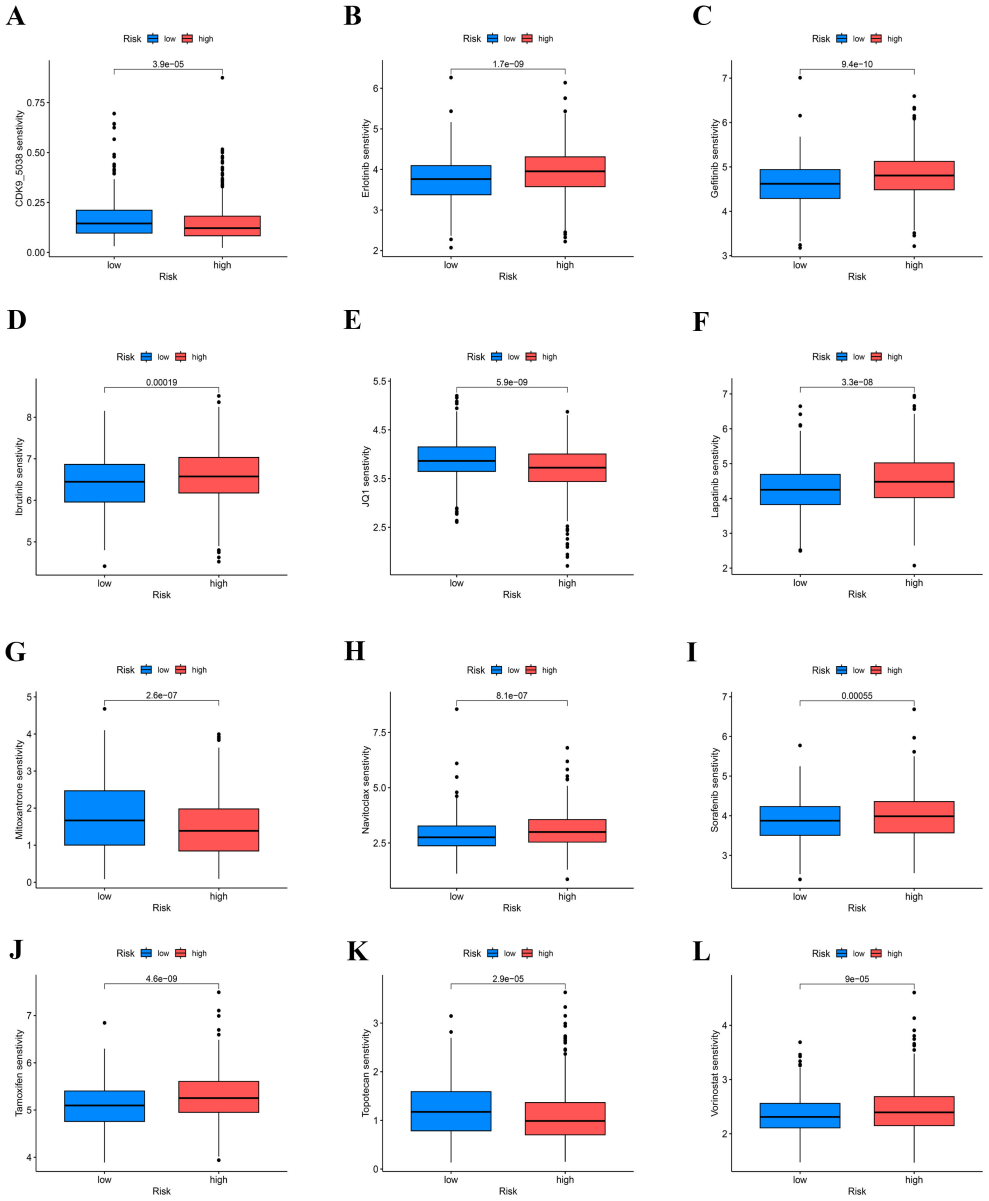
Prediction of COAD patients’ sensitivity to chemotherapeutic drugs. **(A–L)** IC50 values of patients in the high-low risk group.

Additionally, by utilizing the GDSC database, we forecasted the responsiveness of 198 drugs concerning the two risk cohorts. Within this analysis, 52 drugs exhibited varying degrees of sensitivity based on the risk stratification of the cohorts (**Figures 8A–L**). Drugs that are more sensitive to high-risk groups and have therapeutic significance are selected and shown in **Figure 8**. Studies have found that potent and selective CDK9 inhibitors target transcriptional regulation in triple-negative breast cancer (35). Mitoxantrone and gemcitabine are effective in the treatment of metastatic breast cancer (36). JQ-1 (carboxylic acid), BET bromine domain inhibitors have a strong killing effect on triple-negative breast cancer cells (37). Topotecan is useful in lung cancer (38).

### 3.8 Biological function and ASNS expression in melanoma are confirmed

**Figure 9:**
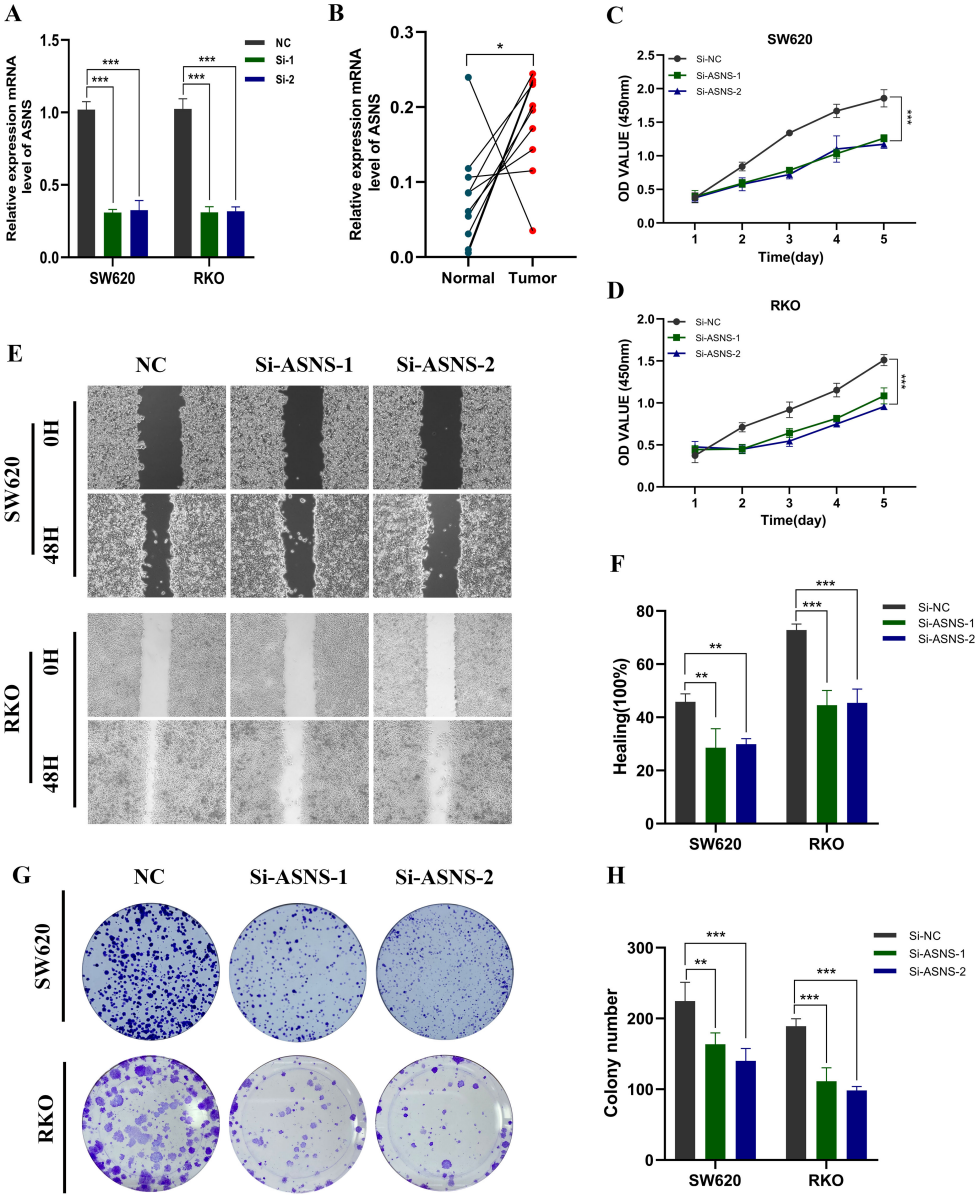
*In vitro* experiment about ASNS. **(A)** Transfection efficiency of ASNS gene. **(B)** Expression level of ASNS gene in tissues. **(C, D)** CCK-8. After ASNS knockdown, the proliferative ability of SW620 and RKO cell lines decreased significantly. **(E, F)** Healing test. After ASNS knockdown, the migration ability of SW620 and RKO cell lines decreased significantly. **(E, F)** Healing test. After ASNS knockdown, the migration ability of SW620 and RKO cell lines decreased significantly. (G, H) Clone formation. After ASNS knockdown, the proliferative ability of the two cell lines decreased significantly. (*p<0.05, **p<0.01, ***p<0.001, ****p<0.0001.)

We knock down the ASNS gene in both SW620 and RKO cells. **Figure 9A** shows the transfection efficiency of the two cell types. To verify the expression level of ASNS in 10 pairs of tissues, the results showed that ASNS was highly expressed in colon cancer tissues (**Figure 9B**). Following in vitro testing, we gained additional insights into the function of ASNS. The CCK-8 study observed a notable decrease in proliferative activity in ASNS knockdown cells (**Figures 9C, D**). Similarly, the healing and migration ability of the examined cell lines were notably diminished following ASNS knockdown (**Figures 9E, F**). Moreover, the colony formation experiments indicated a significant reduction in the proliferation capacity of colon cancer cells after ASNS knockdown (**Figures 9G, H**).

## 4 Discussion

Colon adenocarcinoma represents a prevalent malignant digestive tract tumor, posing a significant threat to human health due to its heterogeneity (39). With the rapid development of medical technology, precision diagnosis, and treatment have become an important focus and have developed rapidly (40). The development of personalized treatment strategies requires an in-depth understanding of the molecular characteristics of COAD to provide patients with more effective and personalized treatment options (41). On the road to exploring COAD precision diagnosis and treatment, the application of various biomarkers and technologies continues to lead the innovation and optimization of diagnosis and treatment strategies (42).

We utilized univariate Cox regression to identify 12 ubiquitin-related genes with prognostic significance. USP26, a member of the specific ubiquitin protease family, is closely associated with tumorigenesis, development, and other pathological processes due to its abnormal regulation (43). MYC, a broad-acting transcription factor, modulates cell differentiation and proliferation through various mechanisms, including transcriptional expansion of target genes (44).

O-linked N-acetylglucosamine transferase (OGT) is a key protein in post-translational modification of O-linked n-acetylglucosamine (O-GlcNAc) that regulates multiple biological processes by linking GlcNAc of glycosyl donors to protein Ser/Thr residues (45). Protein arginine methyltransferase 1 (PRMT1), a member of the arginine methyltransferase family, is often a marker of transcriptional activation and is involved in gene transcription control, mRNA splicing, protein stability regulation, DNA damage signaling, and cell fate determination (46). SNAI1, a nuclear protein, plays a crucial role in the induction of epithelial-mesenchymal transition, formation, and sustenance of embryonic mesoderm, growth arrest, as well as the regulation of cell survival and migration (47). RPS17 is a large RNA molecule that codes for a protein that plays an important biological function in cells and is also a drug target (48). Ribosome binding glycoprotein 2 (RPN2) is a highly conserved glycoprotein that is localized primarily in the rough endoplasmic reticulum (49). Acetyl-CoA carboxylase 1 is a protein encoded by the ACACA gene in the human body. Catalyze rate-limiting reactions during the biological production of long-chain fatty acids (50). RNF112 belongs to the RNF1 family, which includes a variety of transcription factors that play an important role in the regulation of intracellular signaling pathways (51). In a variety of diseases, RNF112 has been shown to have important biological functions (52). Melanocortin 1 receptor (MC1R) is an important gene controlling melanin synthesis in animals (53). The protein encoded by the FBXO39 gene is a member of the F-box protein family and plays a role in regulating protein degradation in cells (54). FBXO39 interacts with the SCF (Skp1-Cullin-F-box) complex through its F-box structure to mediate the ubiquitination degradation of waste proteins (55). Asparagine synthase (ASNS) is a key enzyme in endogenous *de novo* biosynthesis of asparagine (56). ASNS expression is significantly up-regulated in a variety of human tumors, including liver cancer, lung cancer, and other malignant tumors (57). By activating the expression of oncogene KRAS, ASNS promotes the malignant proliferation of tumor cells and leads to tumor progression (58). In our study, ASNS was also found to be a potential target for COAD.

Our study's significant findings hold profound clinical implications. By employing Lasso regression to identify ubiquitination-related gene features in colon adenocarcinoma (COAD), the researchers were able to calculate individualized risk scores for each patient. This risk score not only serves as a tool for risk stratification but also assists clinicians in devising personalized treatment plans to optimize patient outcomes. Moreover, immunological analyses revealed marked differences in immune infiltration levels between the high-risk and low-risk groups. Although the high-risk group demonstrated increased expression of immune checkpoint-related genes, it also exhibited lower microsatellite instability. This may suggest that the tumor microenvironment of the high-risk group possesses characteristics that suppress immune responses. These findings could provide crucial insights for the development of immunotherapy strategies, particularly for high-risk patients, emphasizing the need to address immune evasion mechanisms to formulate more effective therapeutic approaches (59, 60). The single-cell analysis further enables a comprehensive understanding of gene expression across different cell types within the identified features, aiding in the elucidation of COAD's heterogeneity and the complexity of its immune microenvironment. This cell-level analysis lays the groundwork for identifying potential therapeutic targets, especially through the exploration of the ASNS gene, which demonstrates a critical role in COAD. This indicates that ASNS may not only function as a biomarker but also emerge as a novel target for therapeutic intervention.

While immunotherapy has shown initial success in various solid tumors and is a groundbreaking advancement in cancer treatment, its application in COAD is limited, and our comprehension of the COAD immune microenvironment remains inadequate. Consequently, further research on the immune microenvironment of COAD is crucial for advancing immunotherapy. The study revealed higher expression of immune checkpoint-related genes in high-risk COAD patients, along with lower microsatellite instability. This insight can serve as a basis for COAD immune stratification and guide COAD immunotherapy.

## 5 Conclusions

In summary, ubiquitin-associated prognostic markers in COAD facilitate robust patient stratification and comprehensive immunological evaluation. This research holds the potential to inspire novel strategies for COAD detection and therapeutic intervention.

## Data Availability

The datasets presented in this study can be found in online repositories. The names of the repository/repositories and accession number(s) can be found in the article/**Supplementary Material**.

## Supplementary material

Supplementary Figure 1 Volcano plot of the differentially expressed genes.

Supplementary Figure 2 Heatmap of the differentially expressed genes.

Supplementary Figure 3 Ubiquitination-related prognostic genes derived from univariate Cox regression analysis.

Supplementary Figure 4 A heatmap of model gene expression.
