# Supplementary figures and images for: Multi-omics analyses were combined to construct ubiquitination-related features in colon adenocarcinoma and identify ASNS as a novel biomarker
Source: Front Immunol. 2024 Oct 9;15:1466286. doi: 10.3389/fimmu.2024.1466286 (PMC11496147; doi:10.3389/fimmu.2024.1466286)

# Volcano

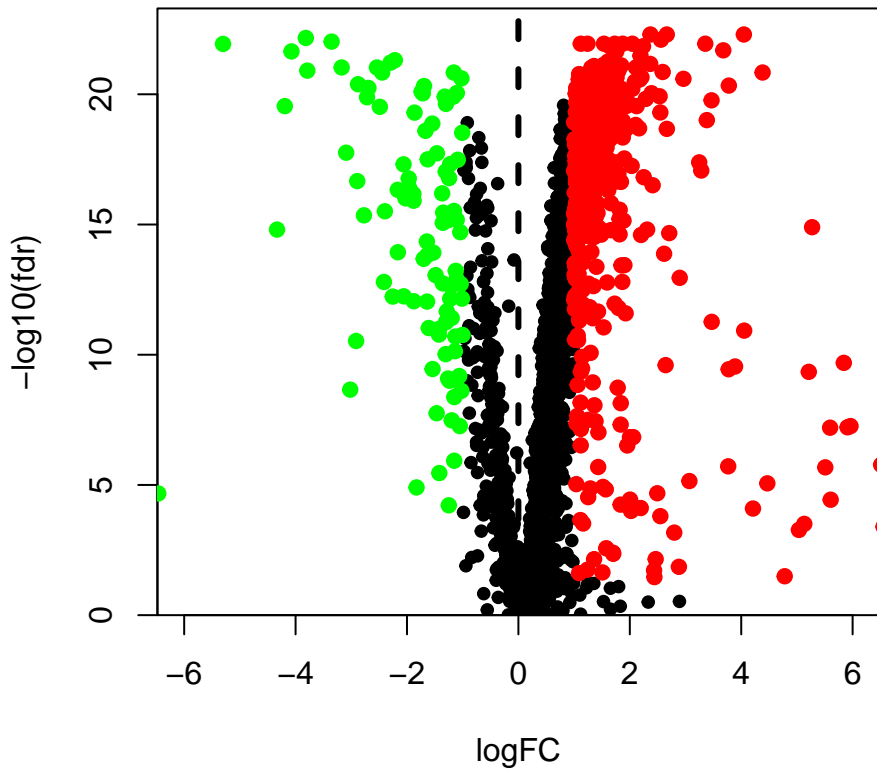

Supplement: Supplementary Figure 1 — Volcano plot of the differentially expressed genes. [file DataSheet1.pdf]

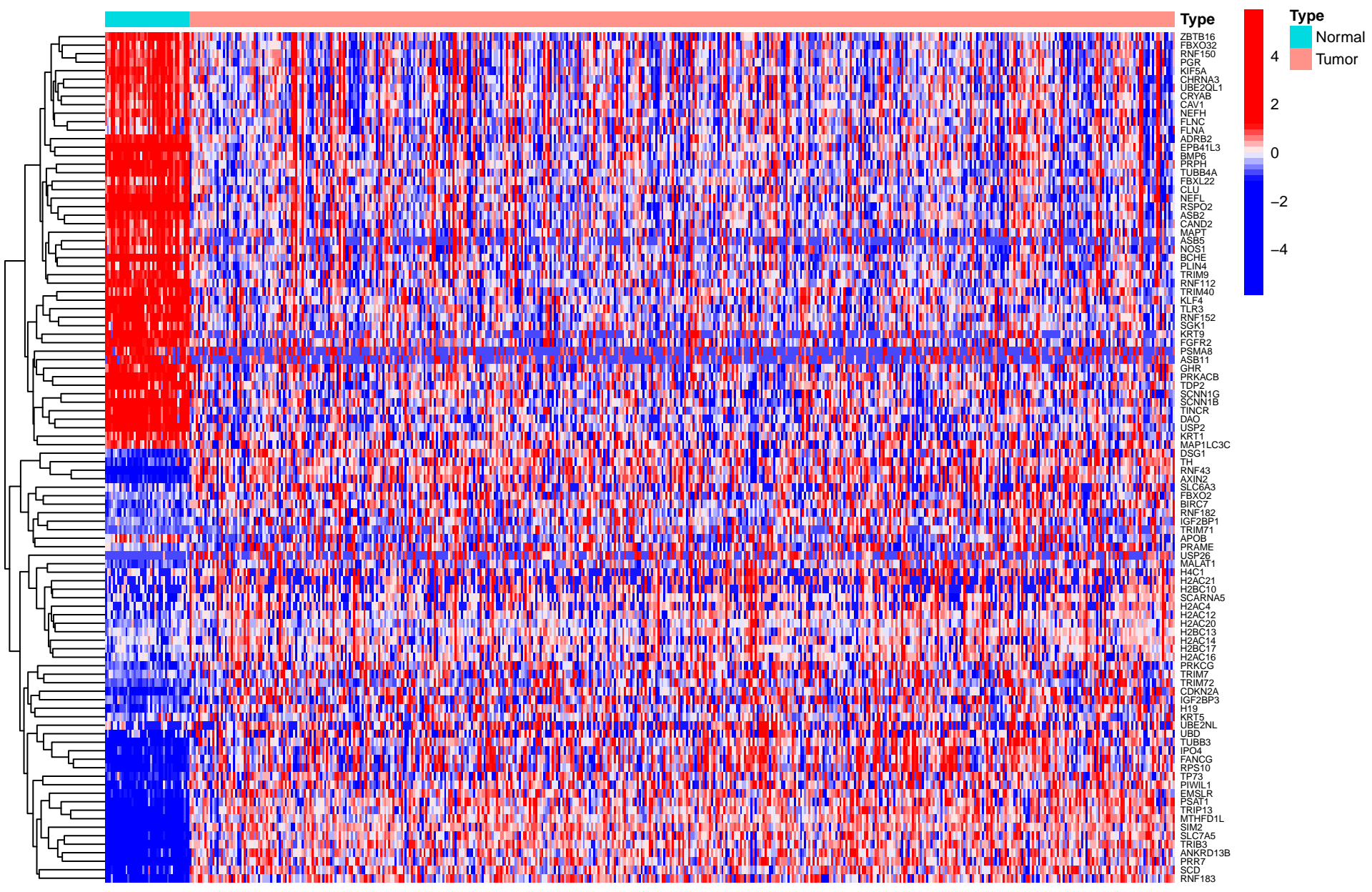

Supplement: Supplementary Figure 2 — Heatmap of the differentially expressed genes. [file DataSheet2.pdf]

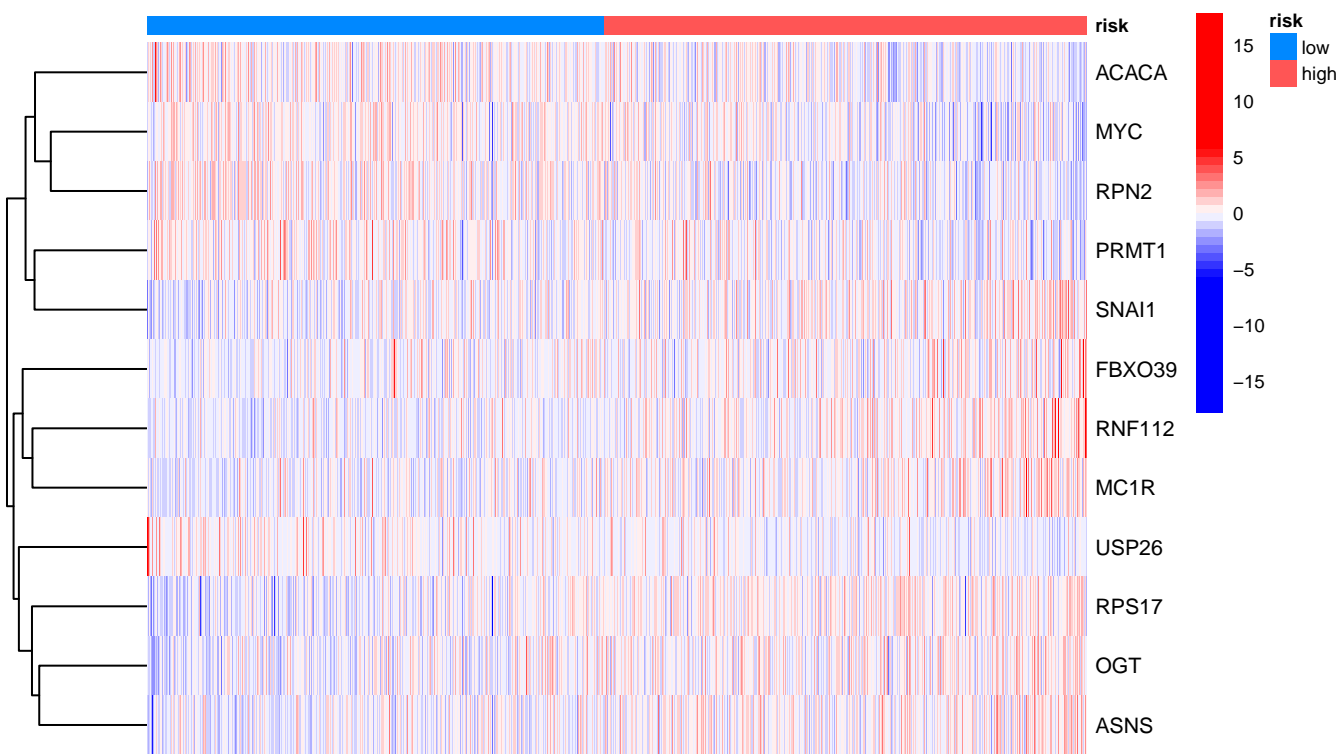

Supplement: Supplementary Figure 4 — A heatmap of model gene expression. [file DataSheet4.pdf]
