# Supplementary material for: Multi-omics analyses were combined to construct ubiquitination-related features in colon adenocarcinoma and identify ASNS as a novel biomarker
Source: Front Immunol. 2024 Oct 9;15:1466286. doi: 10.3389/fimmu.2024.1466286 (PMC11496147; doi:10.3389/fimmu.2024.1466286)
Supplement: Supplementary Figure 3 — Ubiquitination-related prognostic genes derived from univariate Cox regression analysis. [file DataSheet3.pdf]

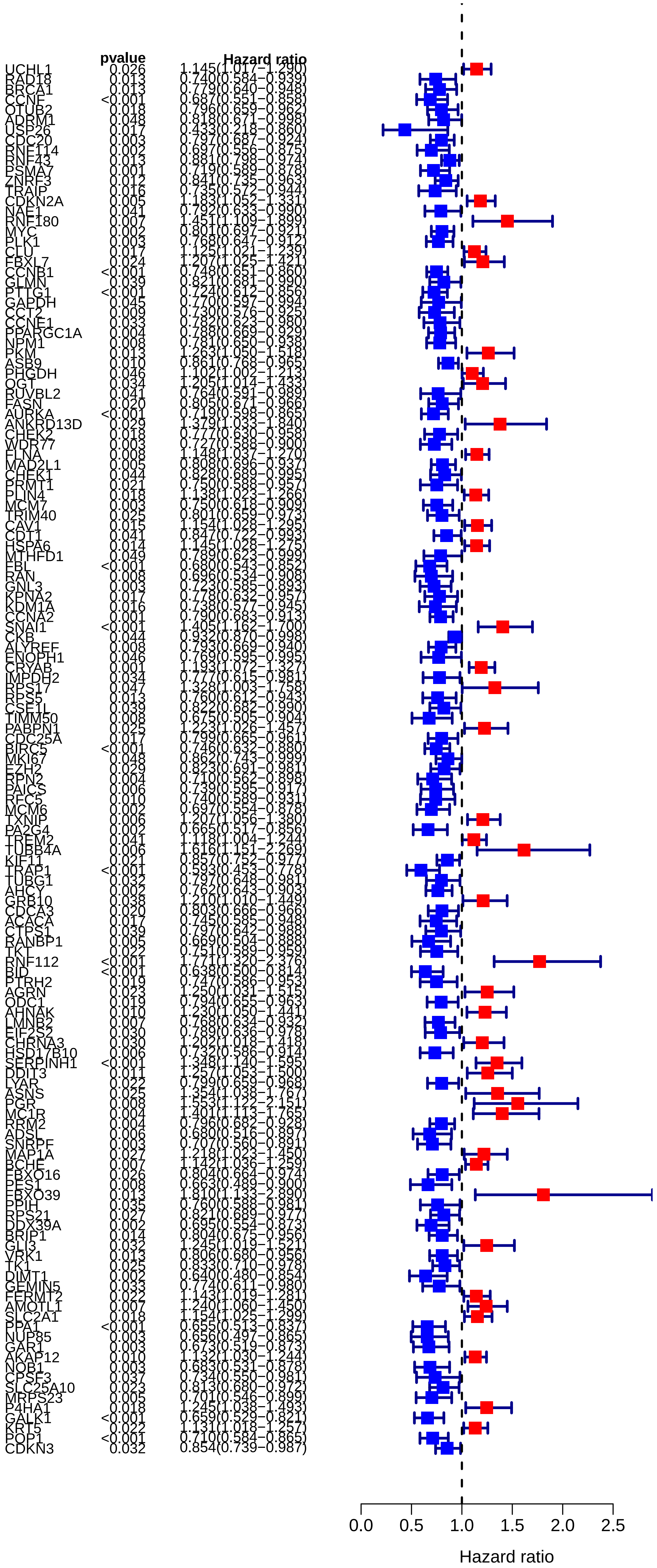
pvalue
Hazard ratio
UCHL1 0.026 1.145(1.017−1.290)
RAD18 0.013 0.740(0.584−0.939)
BRCA1 0.013 0.779(0.640−0.948)
CCNF <0.001 0.687(0.551−0.858)
OTUB2 0.018 0.796(0.659−0.962)
ADRM1 0.048 0.818(0.671−0.998)
USP26 0.017 0.433(0.218−0.860)
CDC20 0.003 0.797(0.687−0.924)
RNF114 0.002 0.697(0.556−0.875)
RNF43 0.013 0.881(0.798−0.974)
PSMA7 0.001 0.719(0.589−0.878)
ZNRF3 0.012 0.841(0.735−0.963)
TRAIP 0.016 0.735(0.572−0.944)
CDKN2A 0.005 1.183(1.052−1.331)
NAE1 0.041 0.792(0.633−0.990)
RNF180 0.007 1.451(1.109−1.899)
MYC 0.002 0.801(0.697−0.921)
PLK1 0.003 0.768(0.647−0.912)
CLU 0.017 1.125(1.021−1.239)
FBXL7 0.024 1.207(1.025−1.421)
CCNB1 <0.001 0.748(0.651−0.860)
GLMN 0.039 0.821(0.681−0.990)
PTTG1 <0.001 0.724(0.612−0.856)
GAPDH 0.045 0.770(0.597−0.994)
CCT2 0.009 0.730(0.576−0.925)
CCNE1 0.033 0.782(0.623−0.980)
PPARGC1A 0.004 0.788(0.669−0.929)
NPM1 0.008 0.781(0.650−0.938)
PKM 0.013 1.263(1.050−1.518)
ASB9 0.010 0.861(0.768−0.965)
PHGDH 0.046 1.102(1.002−1.213)
OGT 0.034 1.205(1.014−1.433)
RUVBL2 0.041 0.764(0.591−0.989)
FASN 0.020 0.805(0.671−0.966)
AURKA <0.001 0.719(0.598−0.865)
ANKRD13D 0.029 1.379(1.033−1.840)
CHEK2 0.018 0.777(0.630−0.958)
WDR77 0.003 0.727(0.588−0.900)
FLNA 0.008 1.148(1.037−1.270)
MAD2L1 0.005 0.808(0.696−0.937)
CHEK1 0.044 0.828(0.689−0.995)
PRMT1 0.021 0.750(0.588−0.957)
PLIN4 0.018 1.138(1.023−1.266)
MCM7 0.003 0.750(0.618−0.909)
TRIM40 0.025 0.801(0.659−0.973)
CAV1 0.015 1.154(1.028−1.295)
CDT1 0.041 0.847(0.722−0.993)
HSPA6 0.014 1.145(1.028−1.275)
MTHFD1 0.049 0.789(0.623−0.999)
FBL <0.001 0.680(0.543−0.852)
RAN 0.008 0.696(0.534−0.908)
GNL3 0.003 0.723(0.585−0.893)
KPNA2 0.017 0.778(0.632−0.957)
KDM1A 0.016 0.738(0.577−0.945)
CCNA2 0.001 0.790(0.683−0.913)
SNAI1 <0.001 1.405(1.162−1.700)
CKB 0.044 0.932(0.870−0.998)
ALYREF 0.008 0.793(0.669−0.940)
ENOPH1 0.046 0.769(0.595−0.995)
CRYAB 0.001 1.193(1.072−1.327)
IMPDH2 0.034 0.777(0.615−0.981)
RPS17 0.047 1.328(1.003−1.758)
RPS5 0.013 0.760(0.612−0.943)
CSE1L 0.039 0.822(0.682−0.990)
TIMM50 0.008 0.675(0.505−0.904)
PABPN1 0.025 1.223(1.026−1.457)
CDC25A 0.017 0.799(0.665−0.961)
BIRC5 <0.001 0.746(0.632−0.880)
MKI67 0.048 0.862(0.743−0.999)
EZH2 0.029 0.823(0.691−0.981)
RPN2 0.004 0.710(0.562−0.898)
PAICS 0.006 0.739(0.595−0.917)
RFC5 0.010 0.740(0.589−0.931)
MCM6 0.002 0.697(0.554−0.878)
TXNIP 0.006 1.207(1.056−1.380)
PA2G4 0.002 0.665(0.517−0.856)
TREM2 0.041 1.118(1.004−1.244)
TUBB4A 0.006 1.616(1.151−2.269)
KIF11 0.021 0.857(0.752−0.977)
TRAP1 <0.001 0.593(0.453−0.778)
TUBG1 0.032 0.797(0.648−0.981)
AHCY 0.002 0.762(0.643−0.903)
GRB10 0.038 1.210(1.010−1.449)
CDCA3 0.020 0.803(0.666−0.966)
ACACA 0.017 0.745(0.585−0.948)
CTPS1 0.039 0.797(0.642−0.988)
RANBP1 0.005 0.669(0.504−0.888)
TKT 0.022 0.751(0.589−0.959)
RNF112 <0.001 1.771(1.320−2.376)
BID <0.001 0.638(0.500−0.814)
PTRH2 0.019 0.747(0.586−0.953)
AGRN 0.023 1.250(1.031−1.515)
ODC1 0.019 0.794(0.655−0.963)
AHNAK 0.010 1.230(1.050−1.441)
LMNB2 0.007 0.768(0.634−0.932)
EIF2S2 0.030 0.789(0.636−0.978)
CHRNA3 0.030 1.202(1.018−1.418)
HSD17B10 0.006 0.732(0.586−0.914)
SERPINH1 <0.001 1.348(1.140−1.595)
DDIT3 0.011 1.257(1.053−1.500)
LYAR 0.022 0.799(0.659−0.968)
ASNS 0.025 1.354(1.038−1.767)
PGR 0.008 1.553(1.122−2.151)
MC1R 0.004 1.401(1.113−1.765)
RRM2 0.004 0.796(0.682−0.928)
ADSL 0.006 0.680(0.516−0.897)
SNRPF 0.003 0.707(0.560−0.891)
MAP1A 0.027 1.218(1.023−1.450)
BCHE 0.007 1.142(1.036−1.259)
FBXO16 0.026 0.804(0.664−0.974)
PES1 0.008 0.663(0.489−0.900)
FBXO39 0.013 1.810(1.133−2.890)
PPIH 0.035 0.760(0.588−0.981)
RPS21 0.027 0.821(0.689−0.977)
DDX39A 0.002 0.695(0.554−0.873)
BRIP1 0.014 0.804(0.675−0.956)
GLI3 0.032 1.245(1.019−1.521)
VRK1 0.013 0.806(0.680−0.956)
TK1 0.025 0.833(0.710−0.978)
DIMT1 0.002 0.640(0.480−0.854)
GEMIN5 0.033 0.774(0.611−0.980)
FERMT2 0.022 1.143(1.019−1.281)
AMOTL1 0.007 1.240(1.060−1.450)
SLC2A1 0.018 1.154(1.025−1.299)
PPA1 <0.001 0.655(0.513−0.837)
NUP85 0.003 0.656(0.497−0.865)
GAR1 0.003 0.673(0.519−0.873)
AKAP12 0.010 1.132(1.030−1.244)
NOB1 0.003 0.683(0.531−0.878)
CPSF3 0.037 0.734(0.550−0.981)
SLC25A10 0.023 0.813(0.680−0.972)
MRPS23 0.005 0.701(0.546−0.899)
P4HA1 0.018 1.245(1.038−1.493)
GALK1 <0.001 0.659(0.529−0.821)
KRT5 0.022 1.131(1.018−1.257)
POP1 <0.001 0.710(0.584−0.865)
CDKN3 0.032 0.854(0.739−0.987)
0.0 0.5 1.0 1.5 2.0 2.5
Hazard ratio
