# Supplementary material for: Multi-omics analyses were combined to construct ubiquitination-related features in colon adenocarcinoma and identify ASNS as a novel biomarker
Source: Front Immunol. 2024 Oct 9;15:1466286. doi: 10.3389/fimmu.2024.1466286 (PMC11496147; doi:10.3389/fimmu.2024.1466286)
Supplement: Supplementary file 5 [file Table1.docx]

| **Oligonucleotides** | **Nucleotide sequence (5'-3')** |
| --- | --- |
| **siRNA** |  |
| Si-ASNS-1 | CGAGTGAAGAAATATCCGTAT |
| Si-ASNS-2 | GCTGTATGTTCAGAAGCTAAA |
|  |  |
| **Primer** |  |
|  |  |
|  |  |
| ASNS | CGCCCAGATTTTCTTCAATCACA (forward) |
|  | TTCCAAACAGCGGGTCAACT (reverse) |
|  |  |

**Table S1. Oligonucleotides used in research**
